# Use of and attitudes to a hospital information system by medical secretaries, nurses and physicians deprived of the paper-based medical record: a case report

**DOI:** 10.1186/1472-6947-4-18

**Published:** 2004-10-16

**Authors:** Hallvard Lærum, Tom H Karlsen, Arild Faxvaag

**Affiliations:** 1 INM, Faculty of Medicine, NTNU, Trondheim, Norway; 2 Sørlandet Sykehus HF Arendal, Arendal, Norway

## Abstract

**Background:**

Most hospitals keep and update their paper-based medical records after introducing an electronic medical record or a hospital information system (HIS). This case report describes a HIS in a hospital where the paper-based medical records are scanned and eliminated. To evaluate the HIS comprehensively, the perspectives of medical secretaries and nurses are described as well as that of physicians.

**Methods:**

We have used questionnaires and interviews to assess and compare frequency of use of the HIS for essential tasks, task performance and user satisfaction among medical secretaries, nurses and physicians.

**Results:**

The medical secretaries use the HIS much more than the nurses and the physicians, and they consider that the electronic HIS greatly has simplified their work. The work of nurses and physicians has also become simplified, but they find less satisfaction with the system, particularly with the use of scanned document images.

**Conclusions:**

Although the basis for reference is limited, the results support the assertion that replacing the paper-based medical record primarily benefits the medical secretaries, and to a lesser degree the nurses and the physicians. The varying results in the different employee groups emphasize the need for a multidisciplinary approach when evaluating a HIS.

## Background

Hospital information systems (HIS) and Electronic Medical Records (EMRs) are considered prerequisites for the efficient delivery of high quality health care in hospitals. However, a large number of legal and practical constraints influence on the design and introduction of such systems [1]. Hence, many EMR implementation projects do not aim at introducing the EMR and eliminating the paper-based counterpart in one step [2]. As a start, the EMR is introduced along with its paper-based counterpart, and both are kept updated. In such environments, health care workers have to deal with a hybrid electronic and paper-based solution. This probably limits the use of EMR [2]. Furthermore, errors are prone to develop due to cumbersome maintenance of the medical record information in dual storage media [3]. In Norway and in other countries, most hospital EMR projects have not passed beyond this phase [1]

Aust-Agder Hospital is the first hospital in Norway to eliminate the paper-based medical record, using a widespread [2] and commercially available HIS in combination with scanning technology. In a recent report, we have evaluated the EMR part of the HIS in this hospital [4], discussing the views of the physicians only. However, to get a more complete picture of the impact of the system, its use by employees other than physicians needs to be evaluated. Both medical secretaries and nurses are important users of a HIS, utilizing both the EMR and the administrative part of the system. The medical secretaries work as transcriptionists, receptionists and coordinators of patient logistics and communication, and the nurses have their own documentation and administrative routines. The elimination of the paper-based medical records is a radical change in the work routines in the hospital organization. To assess the impact of this change on the organization, the EMR system may be described from the perspectives of three important employee groups separately. In this report, we have used questionnaires and interviews to assess how often medical secretaries, nurses and physicians use the HIS system for essential tasks, how easily these tasks are performed using the system, and how satisfied the hospital employees are with it.

## Methods

### The hospital

The investigation was performed in a 410-bed community hospital in Aust-Agder county, Norway. The hospital serves a population of 102,000, caring for 18,600 inpatients and 74,000 outpatients per year (1998). The patients are admitted by primary care physicians external to the hospital and followed up by the hospital physicians. The hospital comprises of departments for psychiatry, general surgery, internal medicine, orthopaedics, gynecology, ear, nose and throat and ophthalmology. Well funded, and with a strong commitment by the hospital administration, the hospital staff began implementation of DIPS 2000^® ^, a commercially available combined EMR and hospital administrative system in March 2000. In April 2001, all except the psychiatric department started to scan documents. From this date, all new patient data was channeled into the EMR in these departments, either as electronic text and data or as scanned documents. The HIS was available in 1100 terminals throughout the hospital, except for the inpatients' rooms. The transition to HIS was administered by a project group, which had been recruited from the hospital staff. The group worked in conjunction with the IT department and the HIS vendor, and was also responsible for communicating with and training the users. The group regularly held series of mandatory hands-on training classes adapted to each profession (3–8 h in total). However, a substantial proportion of the users never attended the classes, particularly the physicians. To reach these users, a task force of medical secretaries was trained and employed during the first month after implementation of the HIS for ambulant training in the wards. Further support was provided by a network of super users (the most experienced users) among the ward staff.

### The EMR

**Figure 1:**
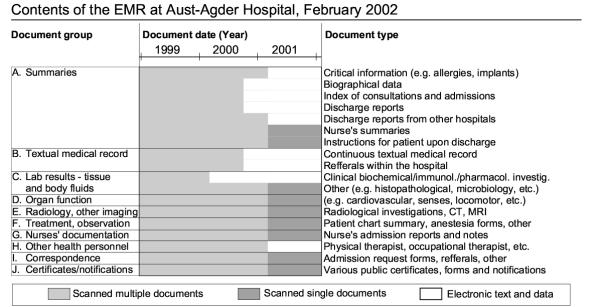
**Contents of the EMR. **Document and information types found in the EMR part of the HIS. Most documents created prior to the implementation of the HIS appear as scanned multiple documents, but some old data has been imported from existing systems and hence appears as electronic text and data. Adapted from Laerum et al [4].

**Figure 2:**
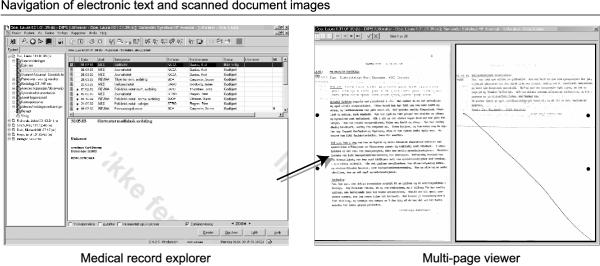
**Navigation of the EMR. **The medical record explorer and the multi-page viewer. Adapted from Laerum et al [4] and reproduced with permission from DIPS ASA, Norway.

The patient data in the EMR part of the HIS is either stored as searchable text and numbers or as document images. The former, called "regular electronic data", essentially consists of the chronological, text-based medical record integrated with lab data in numerical form and textual radiology reports (fig 1). The latter is divided by structure into two categories, as follows: Upon admittance or consultation, the documents in the old paper-based medical records are scanned into the system as digital images in TIFF format. Each image contains all the sheets of one main section of the paper-based record, and hence corresponds to a whole document group (groups A-J in fig 1). These images are called "scanned multiple documents". Upon patient discharge, various paper sheets accumulated during the stay (e.g. the medical treatment form, printouts from diagnostic devices) are scanned, dated and labeled by document type singularly (fig 1). The resulting images are called "scanned single documents". In summary, the patient data is stored as regular electronic data, scanned multiple documents and scanned single documents. They all appear in the hierarchical list in the "medical record explorer" window (fig 2), but are treated separately in this paper, due to their difference in structure, indexation and functionality. The user interface of the HIS system is identical to all types of users, although medical secretaries, nurses and physicians often utilize different parts of system.

### The survey

A questionnaire previously used in a national survey of hospital physicians [2] was modified for this study. The original questionnaire contained sections regarding frequency of use of an EMR system or HIS for specified tasks, user satisfaction with the system as a whole [5] as well as detailed aspects of it [6], and availability of computers. To make the questionnaire applicable to medical secretaries and nurses, new versions of the section regarding frequency of use of the HIS were developed. In collaboration with the authors, 3–6 representatives from the medical secretaries and the nurses identified work tasks for the questionnaire each in two 2-hour group sessions, using recently developed detailed work-flow charts as templates (not shown). The identified tasks were then reduced to 23 and 19 tasks supported by the HIS, respectively (see appendix A). The questionnaire was reviewed in similar sessions by representatives from the physicians. As a result, one new task was added to the physicians' questionnaire, and four tasks not supported by the HIS were removed. For all professions, a new section was added, containing questions about ease of performing each task using the system.

The survey was conducted during February–April 2002, and 85 medical secretaries, 235 nurses and 80 physicians in the medical, surgical and other somatic wards received the questionnaire. Of these, 79 medical secretaries (93%), 172 nurses (73%) and 70 physicians (88%) responded, giving a total response rate of 81% (321/400). We used Teleform™ for data acquisition and SPSS 11.0 for Windows™ for statistical analysis.

In addition to the survey, one of the authors interviewed 8–12 representatives of each profession for 0.5–2 hours. Comments on advantages and disadvantages of the system in all relevant work tasks were noted and summarized

## Results

**Figure 3:**
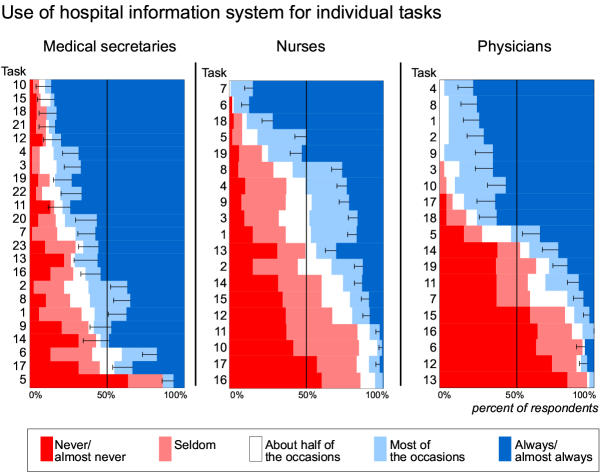
**Use of the Hospital Information System. **Frequency of use of HIS for tasks specific to each profession. Within each profession, the tasks are sorted in descending order by frequency of use. High and low frequency of use is represented by blue and red color tones, respectively. The definitions of the tasks for each profession are given in appendix A. The error bars show the confidence interval of the proportion of respondents answering "Always or almost always".

The medical secretaries used the HIS routinely for most of their tasks defined in the questionnaire. This stands in contrast to the nurses and the physicians (fig 3). The number of tasks with a median response of "always or almost always" was highest for the medical secretaries (15 out of 23 tasks, 65%), and lowest for the nurses (4 out of 19 tasks, 21%).

**Figure 4:**
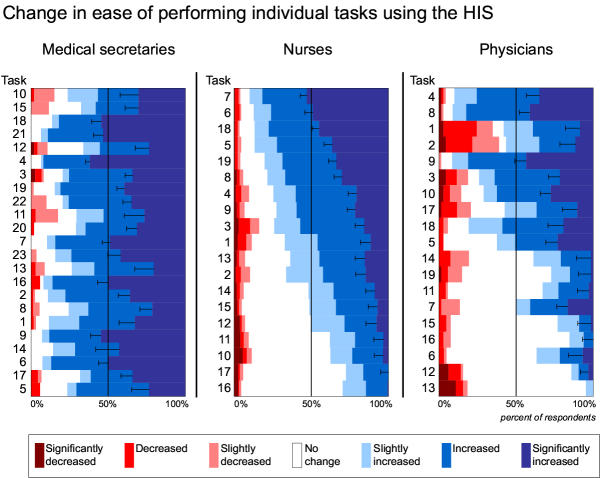
**Task performance using the HIS. **Change in ease of performing individual tasks for each profession when using the HIS. The tasks appear in the same sequence as that of figure 3, i.e. the frequency with which the HIS is used for the task. The responses indicating a task to be easier to perform appear in blue tones, and those indicating it to be more difficult appear in red. The error bars show the confidence interval of the proportion of respondents answering "Significantly increased". For definitions of the individual tasks, see appendix A. (The data for the physicians[4] is included for comparison)

The medical secretaries reported that all of the defined tasks were performed more easily than before the HIS was introduced (i.e. median response for ease of performing the task was "increased" or "significantly increased", in 23 out of 23 tasks, fig 4). In comparison, the number of tasks more easily performed was much lower for the nurses and the physicians (respectively 9 [47%] and 7 [37%] out of 19 individual tasks).

**Figure 5:**
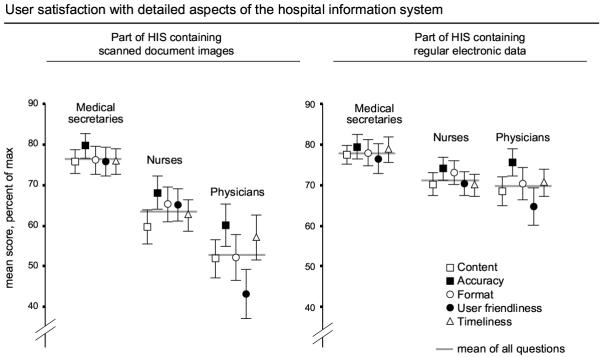
**Detailed user satisfaction. **User satisfaction with detailed aspects of the HIS in various professions. The mean scores of each factor (content, accuracy, format, user friendliness and timeliness) are shown in percent of maximum obtainable score. The error bars show the confidence interval of the mean. (The data for the physicians[4] is included for comparison)

The medical secretaries were much more satisfied with the use of the HIS than the nurses and physicians, both when assessing the detailed aspects of it and the system as a whole. The detailed aspects of the HIS was assessed in twelve questions related to the factors content, accuracy, format, user friendliness and timeliness [6]. The parts of the HIS that contained scanned document images and regular electronic data were assessed separately. The medical secretaries were equally satisfied with both parts of the HIS (fig 5). This stands in contrast to nurses and in particular the physicians, who were less satisfied, particularly with the part containing the scanned document images. The difference between the professions was significant in all factors regarding the scanned document images (ANOVA p < 0.001), and in all factors except accuracy regarding the regular electronic data (fig 5, (ANOVA p = 0.001 to 0.04, p = 0.07 for factor 'accuracy').

**Figure 6:**
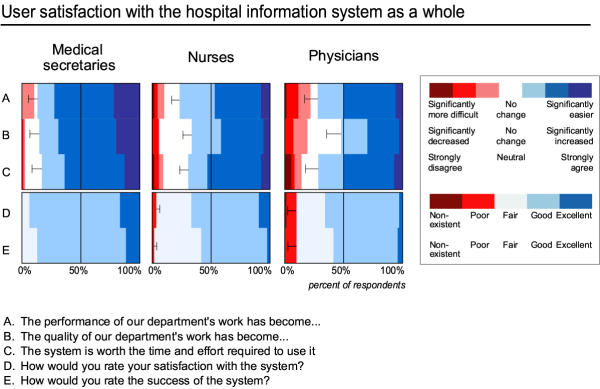
**User satisfaction. **User satisfaction with the HIS as a whole in various professions. The responses colored in red tones represent low satisfaction; those colored in blue tones represent high satisfaction. The error bars show the confidence interval of the combined proportion of all positive responses (The data for the physicians[4] is included for comparison).

In addition to the detailed aspects, the user satisfaction with the HIS as a whole was assessed (fig 6). The medical secretaries gave significantly more positive responses than the nurses and the physicians in all of the five questions in this section (Kruskall-Wallis p = 0.05 in question 2, p < 0.001 in the remaining four questions). However, the majority of each profession gave positive answers in all of these questions. To summarize all results regarding user satisfaction, the system seems to be well adapted to the work of medical secretaries but leave nurses and physicians less satisfied.

**Figure 7:**
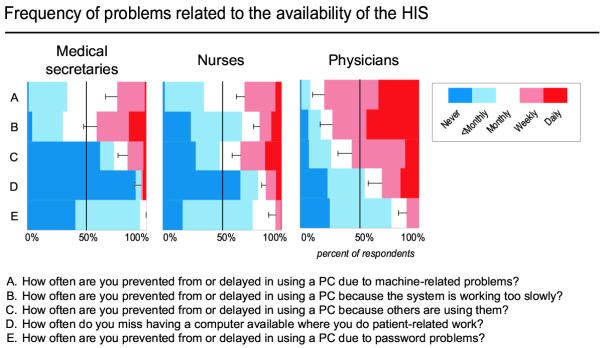
**Problems related to the availability of the HIS. **Reported frequency of problems related to the availability of the HIS. The questions are sorted in descending order by the physicians' frequency of problems. Red tones represent frequent problems, and blue tones represent infrequent problems. The error bars show the confidence interval of the proportion of respondents reporting frequent problems (i.e. weekly or daily).

Partly explaining the differences in user satisfaction, the physicians reported more frequent problems related to availability of the HIS than the medical secretaries and the nurses (fig 7, Kruskall-Wallis p < 0.001 in all questions). The most frequently reported problems among the physicians occurred daily or weekly, and consisted of various software and hardware-related problems, the system working too slowly, and lack of computers where the clinical work was being done. Such problems were not frequently reported among the medical secretaries, except problems with the systems working too slowly (42% daily or weekly, 32/77).

In the interviews, the perceived advantages and disadvantages of the HIS were discussed. Both nurses and physicians in the medical ward found that patient data were more accessible when stored electronically than when stored on paper, in particular regarding lab test data. However, the nurses were still using pen and paper when documenting their activities. The medical secretaries found that generation, handling, fetching and delivery of paper documents and logistics of paper-based patient records had diminished dramatically. The generation of written text had become considerably easier. On the other hand, the scanning process had become an additional burden and was considered time consuming. Overall, handling of paper documents was considered additional work whenever the documents appeared.

## Discussion

In this hospital, we have found that the medical secretaries use the HIS more extensively for their tasks than the nurses and the physicians. Also, they are much more satisfied with the HIS.

Medical secretaries reported that they use the HIS routinely for most of the tasks defined in the questionnaire (fig 3). A simple explanation is that their tasks generally are smaller in scope and have a smaller and more easily defined range of needed information types than that of the nurses and physicians (See appendix A). Hence, the medical secretaries' tasks should be more easily supported by computers than the nurses' and the physicians' tasks. The particular inefficiencies of certain paper-based routines (e.g. regarding task 6, 15, 18 and 19) readily demonstrates the usefulness of computer support [7]. Unlike the work of nurses and physicians, the work of medical secretaries is stationary, avoiding the difficulties in providing an efficient mobile work environment. In addition, each medical secretary typically is assigned a computer, while nurses and physicians usually have to share a limited number of them (fig 7, question C). Another possible reason for the difference in usage pattern could be difference in computer literacy. However, the usage patterns were not consistent with the limited differences found in self-reported computer literacy (data not shown), and the amount of in-house training of medical secretaries and physicians was principally equal.

The medical secretaries reported that all of the tasks in their questionnaire are more easily performed (fig 4). The results from the interviews identify the elimination of the paper-based medical record as a major contributor to this, as several manual paper routines have disappeared (e.g. searching for a lost paper-based medial record or sorting the contents of a medical record) or are replaced by more efficient computer functions (e.g. transferring new lab data to doctors for review). Furthermore, having the administrative functions integrated with the EMR means that a substantial selection of structured demographic, clinical and administrative data is concurrently available to the users of the HIS. This makes several tasks more efficient for the medical secretaries (e.g. sending standard letters to patients in waiting lists). The results are supported by the fact that the number of medical secretaries in the hospital has been reduced by 15 since the onset of the HIS project (Bjørn Engum, personal communication Sept 2003).

Not surprisingly, the medical secretaries were more satisfied with the system than the nurses and the physicians (figs 3 and 4). This agrees with the results of Sittig [8] and Lee [9], who both found that user satisfaction was strongest correlated to questions regarding how easily the work was done. On the other hand, when comparing the user satisfaction scores to the reference data of Doll & Torkzadeh [6], the median user satisfaction score of the medical secretaries lies between the 20^th ^and 30^th ^percentile of the reference data set. This suggests that there is room for improvement of the EMR system regarding the medical secretaries as well as the others. Unlike the nurses and the physicians, the medical secretaries were equally satisfied with the scanned document images as that of the regular electronic medical record. The most likely reason is that the document images are not very often used by the medical secretaries, particularly the document images scanned in sections (data not shown). The disadvantages of the document images, for instance that they can not be searched, therefore seem to affect the user satisfaction of nurses and physicians to a stronger degree than that of the medical secretaries.

The use of the HIS by medical secretaries, nurses and physicians may to some degree be compared at a task-by-task level when the tasks are equally worded. In these tasks, work roles seem to explain the differences. For instance, the tasks "Reviewing the patient's problems" (tasks 1) and "Seek out specific information from patient records" (task 2) appeared in all questionnaires. Of all the respondents, only the physicians had a significant proportion finding that these tasks were more difficult to perform than before (figure 4). A possible reason is that the physicians, in order to perform these tasks as they saw fit for their work role, more often needed to search the scanned document images extensively. When examining the task "Order clinical biochemical laboratory analyses" (task 6 for nurses, task 7 for physicians), the nurses both use the HIS more frequently for this task and find the task more easily to perform than the physicians. However, many Norwegian physicians find that order entry is a task better performed by others [10], reducing the motivation for learning the new system. This way, understanding work roles in the given context appears necessary to interpret the results.

A secondary finding in this study was that the physicians reported frequent computer-related problems, much more frequent than that of medical secretaries and nurses (fig 7). This may be due to escalated demands on computing power, system stability and availability. Without the paper-based medical record, the EMR is taken into full use and the real demands of supporting the physicians' information processing are revealed. The high reported frequency of computer-related problems may partly explain the overall lower user satisfaction of the physicians, as well as the relatively high proportion of physicians finding certain tasks more difficult to perform (task 1 and 2, fig 4). An observational study could elaborate on these relationships, focusing on what kinds of computer problems are the least tolerable to the physicians.

### Limitations of the study

In the questionnaire, we do not know how often each task is carried out (using the HIS or not) or how long it takes, which means that demanding tasks might be outnumbered by the less demanding ones. Furthermore, the list of tasks supported in some way by the system may not be complete, and the list does not cover the full range of conceivable tasks suited for support by any given HIS. However, given that the tasks defined for each group cover important parts of their information-related work, a cautious comparison of general patterns of use between groups of hospital employees is possible.

## Conclusion

Evaluation of a HIS in a hospital that has eliminated the paper-based medical record reveals considerable differences in user satisfaction and reported use of the system among medical secretaries, nurses and physicians. Although the basis for reference is limited, the results seem to support the claim that replacing the paper-based medical record primarily benefits the medical secretaries, and to a lesser degree the nurses and the physicians. Inspired by Aust-Agder Hospital, two of 22 other Norwegian hospitals using the same system (as of Aug 2002) are about to eliminate the paper-based medical record, making a future comparison between hospitals possible. When assessing the effects of a HIS on a hospital organization by asking users, the multidisciplinary nature of health care provision should be reflected in the selection of hospital employees that participate in the evaluation.

## Competing interests

The authors declare that they have no competing interests.

## Authors' contributions

HL, THK and AF planned the investigation. HL and THK developed the questionnaires for the medical secretaries and nurses. THK organized the administration of the questionnaires, and HL scanned and analysed the data. HL, THK and AF wrote the manuscript jointly.

## Pre-publication history

The pre-publication history for this paper can be accessed here:



## Supplementary Material

Additional File 1 *Appendix A: Task lists *The three lists of tasks as they appear in the questionnaire developed for the medical secretaries, nurses and physicians, respectively. Click here for file

Additional File 2 An English translation of the questionnaire used for the physicians in the survey. It is meant for review purposes. Click here for file
