# Supplementary material for: Use of and attitudes to a hospital information system by medical secretaries, nurses and physicians deprived of the paper-based medical record: a case report
Source: BMC Med Inform Decis Mak. 2004 Oct 16;4:18. doi: 10.1186/1472-6947-4-18 (PMC526259; doi:10.1186/1472-6947-4-18)
Supplement: Additional File 1 — Appendix A: Task lists The three lists of tasks as they appear in the questionnaire developed for the medical secretaries, nurses and physicians, respectively. [file 1472-6947-4-18-S1.doc]

# Appendix A Tasks for medical secretaries, nurses and physicians

In this appendix, the tasks appearing in the questionnaires for The tasks are shown in the order they appear in figures 3 and 4, i.e. sorted in descending order by reported frequency of use of the HIS for this task.

## Medical secretaries

| **Task number** | **Task** |
| --- | --- |
| 10 | Register refferals |
| 15 | Send standard note to patient (letter for waiting lists, admissions, preparations for investigations) |
| 18 | Type dictations in textual part of patient record |
| 21 | Make and send discharge notes |
| 12 | Book, rebook or cancel appointments for outpatient clinic or planned admissions |
| 4 | Review the patient's contacts with the hospital (e.g. last admission or appointment in the outpatient clinic) |
| 3 | Locate the patient's paper documents (including paper-based medical record) |
| 19 | Keep the documents in the paper-based or electronic medical record sorted |
| 22 | Select and register codes for diagnoses and performed procedures |
| 11 | Register patients in waiting lists |
| 20 | Type documents that is not part of the running textual medical record (i.e. admission notes, daily notes and discharge notes, f.ex. individual letters, requests) |
| 7 | Obtain information regarding address of patient or relatives |
| 23 | Make or print out reports of aggregated data, patient list, etc. |
| 13 | Coordinate appointments for investigations with appointments in outpatient clinics or planned admissions |
| 16 | Transfer documents for ratification of physician |
| 2 | Seek out specific information from patient records |
| 8 | Obtain information regarding name and address of patient's primary physician or local hospital |
| 1 | Review the patient's problems |
| 9 | Review inpatients in the ward (name, date of birth, room) |
| 14 | Transfer patient to another ward or institution |
| 6 | Obtain or print out results from lab tests, radiological or other supplemental investigations |
| 17 | Transfer documents regarding internal communication in the hospital (e.g. refferals) |
| 5 | Review the patient's list of medications |

## Nurses

| **Task number** | **Task** |
| --- | --- |
| 7 | Obtain results from clinical biochemical laboratory analyses |
| 6 | Order clinical biochemical laboratory analyses |
| 18 | Review patients currently admitted to the ward (name, date of birth, room) |
| 5 | Obtain results from new tests or investigations |
| 19 | Transfer patient to other section, ward or institution |
| 8 | Obtain results from x ray, ultrasound, or CT investigations |
| 4 | Follow results of a test or investigation over time |
| 9 | Obtain results from other supplemental investigations |
| 3 | Seek out specific information from patient records |
| 1 | Review the patient's problems |
| 13 | Keep list of short notes about each patient (e.g. problems and diagnoses) |
| 2 | Follow the patient's condition over time |
| 14 | Collect patient data for daily updates of nurses' documentation |
| 15 | Collect patient data for nurses' summarizing report |
| 12 | Collect patient data for documentation during admission |
| 11 | Check allergies regarding medications |
| 10 | Obtain the patient's medication list |
| 17 | Give written general information to patients |
| 16 | Give written specific information to patients |

## Physicians

| **Task number** | **Task** |
| --- | --- |
| 4 | Obtain results from new tests or investigations |
| 8 | Obtain results from clinical biochemical laboratory analyses |
| 1 | Review the patient's problems |
| 2 | Seek out specific information from patient records |
| 9 | Obtain results from x ray, ultrasound, or CT investigations |
| 3 | Follow results of a test or investigation over time |
| 10 | Obtain results from other supplemental investigations |
| 17 | Collect patient information for discharge reports |
| 18 | Check and sign typed dictations |
| 5 | Enter daily notes |
| 14 | Collect patient data for various medical declarations |
| 19 | Register codes for diagnosis or performed procedures |
| 11 | Refer patient to other departments or specialists |
| 7 | Order clinical biochemical laboratory analyses |
| 15 | Give written specific information to patients |
| 16 | Give written general information to patients |
| 6 | Produce data reviews for specific patient groups |
| 12 | Write prescriptions |
| 13 | Write sick leave notes |
