# Supplementary material for: Use of and attitudes to a hospital information system by medical secretaries, nurses and physicians deprived of the paper-based medical record: a case report
Source: BMC Med Inform Decis Mak. 2004 Oct 16;4:18. doi: 10.1186/1472-6947-4-18 (PMC526259; doi:10.1186/1472-6947-4-18)
Supplement: Additional File 2 — An English translation of the questionnaire used for the physicians in the survey. It is meant for review purposes. [file 1472-6947-4-18-S2.pdf]

Doctors

Check like this: ☒
not like this: ☐

A. About your work position

Clinical position: ☐ Intern ☐ Resident ☐ Consulting physician

B. About your experience with computers

		Yes	No
1	Do you own a computer?	☐	☐

2 How many fingers du you use when typing? ☐ Two ☐ Three or more ☐ All (or touch)

	3 Have you earlier* used a computer for	Yes	No
a)	Test result retrieval	☐	☐
b)	Literature search	☐	☐
c)	Word processing	☐	☐
d)	Entering patient info (e.g. an outpatient note)	☐	☐
e)	Retrieving patient info (e.g. a previous discharge report)	☐	☐

		Never	Less frequently than monthly	Monthly	Weekly	Daily
4	In the past*, what is the most frequent you used a computer?	☐	☐	☐	☐	☐

		Lowest		Average		Highest
5a	How would you rate your computer skills in general?	☐	☐	☐	☐	☐
5b	How would you rate your computer skills concerning DIPS?	☐	☐	☐	☐	☐

* *Before DIPS was installed, or before you started working in the hospital*

C. About the availability of computers at your working place at the hospital

		Yes	No
1	Do you have a computer in your office?	☐	☐

		Never	Less frequently than monthly	Monthly	Weekly	Daily
2	About the computers installed in the ward, at the outpatient clinic offices, investigation rooms, etc: How often are you prevented from or delayed in using them because others are using them?	☐	☐	☐	☐	☐
3	How often do you miss having a computer available where you do patient-related work?	☐	☐	☐	☐	☐
4	How often are you prevented from or delayed in using a computer due to computer errors, system crashes or other machine-related problems?	☐	☐	☐	☐	☐
5	How often are you prevented from or delayed in using a computer due to password problems?	☐	☐	☐	☐	☐
6	How often are you prevented from or delayed in using a computer because the system is working too slowly?	☐	☐	☐	☐	☐

D. About your use of DIPS for clinical tasks in the hospital

At Aust-Agder Hospital an electronic medical record (DIPS 2000) is installed, consisting of "purely" electronic data and scanned paper records. The latter contains mainly older documents from the patient record, but also some newer information (external lab tests, refferals, etc.) Because the part of DIPS handling the scanned information differs so much from the rest of the system, we are keeping it separate in this questionnaire.

D1. When you expect to find the information in the scanned part of DIPS, how often do you use it for the following tasks:

	Never/ almost never	Seldom	About half of the occasions	Most of the occasions	Always/ almost always
1 Review the patient's problems					
a. from documents scanned in sections ("bulk")	☐	☐	☐	☐	☐
b. from documents scanned one sheet at a time	☐	☐	☐	☐	☐
2 Seek out specific information from patient records					
a. from documents scanned in sections ("bulk")	☐	☐	☐	☐	☐
b. from documents scanned one sheet at a time	☐	☐	☐	☐	☐
3 Follow the results of a particular test or investigation over time					
a. from documents scanned in sections ("bulk")	☐	☐	☐	☐	☐
b. from documents scanned one sheet at a time	☐	☐	☐	☐	☐
4 Obtain the results from clinical biochemical laboratory analyses					
a. from documents scanned in sections ("bulk")	☐	☐	☐	☐	☐
b. from documents scanned one sheet at a time	☐	☐	☐	☐	☐
5 Obtain the results from X-ray, ultrasound or CT investigations					
a. from documents scanned in sections ("bulk")	☐	☐	☐	☐	☐
b. from documents scanned one sheet at a time	☐	☐	☐	☐	☐
6 Obtain the results from other supplementary investigations					
a. from documents scanned in sections ("bulk")	☐	☐	☐	☐	☐
b. from documents scanned one sheet at a time	☐	☐	☐	☐	☐
7 Obtain the results from new tests or investigations *(always scanned on sheet at time)*	☐	☐	☐	☐	☐

D2. The scanned document images notwithstanding, how often do you use DIPS to assist you with the following tasks:

		Never/ almost never	Seldom	About half of the occasions	Most of the occasions	Always/ almost always
1	Review the patient's problems	☐	☐	☐	☐	☐
2	Seek out specific information from patient records	☐	☐	☐	☐	☐
3	Follow the results of a particular test or investigation over time	☐	☐	☐	☐	☐
4	Obtain the results from new tests or investigations	☐	☐	☐	☐	☐
5	Enter daily notes	☐	☐	☐	☐	☐
6	Produce data reviews for specific patient groups, e.g. complication rate, complications	☐	☐	☐	☐	☐
7	Order clinical biochemical laboratory analyses	☐	☐	☐	☐	☐
8	Obtain the results from clinical biochemical laboratory analyses	☐	☐	☐	☐	☐
9	Obtain the results from X-ray, ultrasound or CT investigations	☐	☐	☐	☐	☐
10	Obtain the results from other supplementary investigations	☐	☐	☐	☐	☐
11	Refer the patient to other departments or specialists	☐	☐	☐	☐	☐
12	Write prescriptions	☐	☐	☐	☐	☐
13	Complete sick-leave forms	☐	☐	☐	☐	☐
14	Collect patient information for various medical declarations	☐	☐	☐	☐	☐
15	Give written individual information to patients, e.g. about medications, disesase status	☐	☐	☐	☐	☐
16	Give written general medical information to patients	☐	☐	☐	☐	☐
17	Collect patient info for discharge reports	☐	☐	☐	☐	☐
18	Check and sign typed dictations	☐	☐	☐	☐	☐
19	Register codes for diagnosis or performed procedures	☐	☐	☐	☐	☐

20 Other (please specify)

E1. About your satisfaction with DIPS, the part handling the scanned paper records

Here we would like to learn your opinion of the part of DIPS handling the ***scanned*** *paper records*

1 Content	Never/ almost never	Seldom	About half of the time	Most of the time	Always/ almost always
a How often does the system provide the precise information you need?	☐	☐	☐	☐	☐
b How often does the information content meet your needs?	☐	☐	☐	☐	☐
c How often does the system provide reports[1] that seem to be just about exactly what you need?	☐	☐	☐	☐	☐
d How often does the system provide sufficient information?	☐	☐	☐	☐	☐

2 Accuracy [2]	Never/ almost never	Seldom	About half of the time	Most of the time	Always/ almost always
a How often is the system accurate?	☐	☐	☐	☐	☐
b How often are you satisfied with the accuracy of the system?	☐	☐	☐	☐	☐

3 Format	Never/ almost never	Seldom	About half of the time	Most of the time	Always/ almost always
a How often do you think the output is presented in a useful format?	☐	☐	☐	☐	☐
b How often is the information clear?	☐	☐	☐	☐	☐

4 Ease of use	Never/ almost never	Seldom	About half of the time	Most of the time	Always/ almost always
a How often is the system user-friendly?	☐	☐	☐	☐	☐
b How often is the system easy to use?	☐	☐	☐	☐	☐

5 Timeliness	Never/ almost never	Seldom	About half of the time	Most of the time	Always/ almost always
a How often do you get the information you need in time?	☐	☐	☐	☐	☐
b How often does the system provide up-to-date information?	☐	☐	☐	☐	☐

1 *"Report" may be interpreted as a selection or resume of information shown on the screen or printed*
2 *E.g. that correct journal, patient and document type is displayed; that the information (e.g. blod pressure) is presented having the right name; that the values are correct, etc.*

E1. About your satisfaction with DIPS, the part handling the regular electronic data

Here we would like to learn your opinion of DIPS, ***regardless of*** *the part handling the scanned paper records*

1 Content	Never/ almost never	Seldom	About half of the time	Most of the time	Always/ almost always
a How often does the system provide the precise information you need?	☐	☐	☐	☐	☐
b How often does the information content meet your needs?	☐	☐	☐	☐	☐
c How often does the system provide reports that seem to be just about exactly what you need?	☐	☐	☐	☐	☐
d How often does the system provide sufficient information?	☐	☐	☐	☐	☐

2 Accuracy [1]	Never/ almost never	Seldom	About half of the time	Most of the time	Always/ almost always
a How often is the system accurate?	☐	☐	☐	☐	☐
b How often are you satisfied with the accuracy of the system?	☐	☐	☐	☐	☐

3 Format	Never/ almost never	Seldom	About half of the time	Most of the time	Always/ almost always
a How often do you think the output is presented in a useful format?	☐	☐	☐	☐	☐
b How often is the information clear?	☐	☐	☐	☐	☐

4 Ease of use	Never/ almost never	Seldom	About half of the time	Most of the time	Always/ almost always
a How often is the system user-friendly?	☐	☐	☐	☐	☐
b How often is the system easy to use?	☐	☐	☐	☐	☐

5 Timeliness	Never/ almost never	Seldom	About half of the time	Most of the time	Always/ almost always
a How often do you get the information you need in time?	☐	☐	☐	☐	☐
b How often does the system provide up-to-date information?	☐	☐	☐	☐	☐

1 *E.g. that correct journal, patient and document type is displayed; that the information (e.g. blod pressure) is presented having the right name; that the values are correct, etc.*

F. In your opinion, how has DIPS changed the performance of the following tasks in your department:

		Significantly more difficult	More difficult	Slightly more difficult	No change	Slightly easier	Easier	Significantly easier
1	To review the patient's problems has become	☐	☐	☐	☐	☐	☐	☐
2	To seek out specific information from patient records has become	☐	☐	☐	☐	☐	☐	☐
3	To follow the results of a particular test or investigation over time has become	☐	☐	☐	☐	☐	☐	☐
4	To obtain the results from new tests or investigations has become	☐	☐	☐	☐	☐	☐	☐
5	To enter daily notes has become	☐	☐	☐	☐	☐	☐	☐
6	To produce data reviews for specific patient groups (eg. complication rate) has become	☐	☐	☐	☐	☐	☐	☐
7	To order clinical biochemical laboratory analyses has become	☐	☐	☐	☐	☐	☐	☐
8	To obtain the results from clinical biochemical laboratory analyses has become	☐	☐	☐	☐	☐	☐	☐
9	To obtain the results from X-ray, ultrasound or CT investigations has become	☐	☐	☐	☐	☐	☐	☐
10	To obtain the results from other supplementary investigations has become	☐	☐	☐	☐	☐	☐	☐
11	To refer the patient to other departments or specialists has become	☐	☐	☐	☐	☐	☐	☐
12	To write prescriptions has become	☐	☐	☐	☐	☐	☐	☐
13	To complete sick-leave forms has become	☐	☐	☐	☐	☐	☐	☐
14	To collect patient information for various medical declarations has become	☐	☐	☐	☐	☐	☐	☐
15	To give written individual information to patients, (e.g. about medications, disesase status) has become	☐	☐	☐	☐	☐	☐	☐
16	To give written general medical information to patients has become	☐	☐	☐	☐	☐	☐	☐
17	To collect patient info for discharge reports has become	☐	☐	☐	☐	☐	☐	☐
18	To check and sign typed dictations has become	☐	☐	☐	☐	☐	☐	☐
19	The register codes for diagnosis or performed procedures has become	☐	☐	☐	☐	☐	☐	☐

G. Global assessment of DIPS

1 All considered, to what extent has DIPS changed these two aspects of your own department?

	Significantly more difficult	More difficult	Slightly more difficult	No change	Slightly easier	Easier	Significantly easier
a The performance of our department's work has become	☐	☐	☐	☐	☐	☐	☐

	Significantly decreased	Decreased	Slightly decreased	No change	Slightly increased	Increased	Significantly increased
b The quality of our department's work has become	☐	☐	☐	☐	☐	☐	☐

2 How much do you agree with the following statement:

	Strongly disagree	Disagree	Slightly disagree	Neutral	Slightly agree	Agree	Strongly disagree
DIPS is worth the time and effort required to use it	☐	☐	☐	☐	☐	☐	☐

	non-existent	poor	fair	good	excellent
3 All considered, how would you rate your satisfaction with DIPS in your department?	☐	☐	☐	☐	☐

	non-existent	poor	fair	good	excellent
4 All considered, how would you rate the success of DIPS in your department?	☐	☐	☐	☐	☐

H. Comments

In your opinion, are any of the functions in DIPS particularly useful? If so, please explain

In your opinion, are any of the functions in DIPS not very useful? If so, please explain

Do you miss any functionality in DIPS? If so, please explain

Were parts of the questionnaire unclear or ambiguous? Other comments?
